# Development and validation of PART (Pharmacotherapy Assessment in Renal Transplant Patients) criteria to assess drug‐related problems in an outpatient renal transplant population: A cross‐sectional study

**DOI:** 10.1002/prp2.453

**Published:** 2019-01-15

**Authors:** Layal El Raichani, Qian Du, Alexandre Mathieu, Sabrina Almassy, Lyne Lalonde, Djamal Berbiche, Elisabeth Gélinas‐Lemay, Nathalie Boudreau, Héloïse Cardinal

**Affiliations:** ^1^ Department of Pharmacy Centre intégré de santé et de services sociaux CISSS de la Montérégie Centre Longueuil Quebec Canada; ^2^ Faculty of Pharmacy University of Montreal Montreal Quebec Canada; ^3^ Department of Pharmacy Centre intégré universitaire de santé et de services sociaux du Centre‐Sud‐de‐l’île‐Montréal Montreal Quebec Canada; ^4^ Department of Pharmacy Centre Hospitalier de l'Université de Montréal Montreal Quebec Canada; ^5^ Department of Pharmacy Centre intégré universitaire de santé et de services sociaux de l'Estrie Sherbrooke Quebec Canada; ^6^ Faculty of Medicine and Health Sciences Université de Sherbrooke Sherbrooke Quebec Canada; ^7^ Research center Centre Hospitalier de l'Université de Montréal Montreal Quebec Canada; ^8^ Nephrology Division Centre Hospitalier de l'Université de Montréal Montreal Quebec Canada

**Keywords:** drug‐related problems, kidney transplantation, quality improvement

## Abstract

Kidney transplant recipients are at risk of pharmacological interactions and adverse drug reactions. Community pharmacists are uniquely poised to detect and intervene in cases of drug‐related problems. The aims of this study were to develop and validate a list of explicit criteria to be used by community pharmacists to assess drug‐related problems in kidney transplant patients, and to assess their frequency and their determinants. First, we used a modified RAND method where a panel of experts established the PART (Pharmacotherapy Assessment in Renal Transplant Patient) criteria. Then, we performed a cross‐sectional study in which we applied the PART criteria to 97 prevalent kidney transplant recipients followed at a single university‐affiliated center. The final list of PART criteria included 70 drug‐related problems and was reliable (kappa: 0.88). An average of 1.2 drug‐related problems per patient was detected when the PART criteria were applied, with 68% of patients having at least 1 problem. This figure was 1.4 per patient using the expert judgment of renal transplant pharmacists who had no access to the PART list. The total number of medications taken was the only factor associated with the number of drug‐related problems (β: 0.27 for an increase of five medications, 95% CI 0.005, 0.547). The PART criteria provide a novel tool for community pharmacists to systematically detect drug‐related problems in kidney transplant recipients.

**Abbreviations**
CKD: chronic kidney disease
DRPs: drug‐related problems
KTR: kidney transplant recipients
LDL‐C: low‐density lipoprotein cholesterol

## 1 INTRODUCTION

Renal transplantation is the optimal treatment modality for patients with end‐stage renal disease, improving both quality of life and life expectancy compared to dialysis.^1^, ^2^ However, transplantation entails significant changes in pharmacotherapy for patients, with complex medication regimen including multiple immunosuppressive agents that have pharmacokinetic and pharmacodynamic interactions with other drugs, frequent adverse events and a narrow therapeutic index.^3^

After the early post‐transplant period, patients are regularly returned to their referring center and may have limited access to expert transplant pharmacists who would rapidly intervene when problems occur with the medication regimen.^4^ This is particularly relevant as transplant patients are at risk for serious adverse events such as graft rejection, opportunistic infections, hospitalization, and drug toxicity due to errors in their pharmacotherapy.^3^ Furthermore, low adherence remains problematic in many cases, as 20% to 70% of kidney transplant recipients (KTR) are not totally adherent to their immunosuppressant therapy, increasing the risk of rejection.^5^ Hence, the risk of drug‐related problems (DRPs) is elevated in this patient population. A DRP is an event or circumstance involving drug therapy that actually or potentially interferes with desired health outcomes.^6^, ^7^

Community pharmacists are accessible for consultation and follow patients regularly. They are thus uniquely poised to improve care for KTR. Community pharmacists review the pharmacological profile of their patients with each medication renewal and can intervene quickly by notifying the transplant center, the primary care provider or the patient if a DRP arises.^8^ However, given the relative scarcity of organ transplant recipients in the general population and the absence of extensive training on the topic in their curriculum, most community pharmacists lack the expertise necessary to detect DRPs in transplant patients accurately and rapidly.

Although decision tools to help evaluate pharmacotherapy in older adults and in patients with chronic kidney disease (CKD) have been created, 9, 10, 11 no such instrument exists for KTR. Therefore, the aims of this study were to develop and validate a list of explicit criteria to be used by community pharmacists to assess DRPs in KTRs, to determine the number and types of DRPs, and identify factors associated with the presence of DRPs in this patient population. Although future studies will be needed to assess the use of our tool in community pharmacies, here we report the first step of a quality improvement process where the ultimate goal is to improve the quality of pharmacotherapy in KTR while providing community‐based services.

## 2 MATERIALS AND METHODS

### 2.1 Development of the PART criteria

#### 2.1.1 Phase 1

The PART criteria were developed following a modified RAND appropriateness method.^12^ In the first phase, a list of potentially important DRPs for KTR was created through a review of the literature and consultation with transplant nephrologists and pharmacists. A short summary of the available evidence and supporting references were provided for each DRP.

#### 2.1.2 Phase 2

A panel of experts including four transplant nephrologists from two different adult transplant centers in Montreal, four renal transplant pharmacists from four different transplant centers in Montreal and Ottawa, two community pharmacists and two family physicians from the region of Montreal was assembled to evaluate the appropriateness of each potential DRP from the initial list. First, in March 2016, an electronic survey including each DRP and a short bibliography was sent to the panel. The experts were asked to evaluate the clinical appropriateness of each DRP on a Likert scale ranging from 1 to 9 (1 being totally inappropriate and 9 being totally appropriate). Experts were allowed to propose amendments to existing DRPs and propose new DRPs if they so wished. The median score of each DRP was calculated and DRPs were classified as “appropriate” (median score of 7 to 9), “uncertain” (median score of 4 to 6) and “inappropriate” (median score of 1 to 3).

The experts then attended a 4‐hour panel session at the Centre Hospitalier de l'Université de Montréal (CHUM) in May 2016 to determine the final PART criteria. Using data from the initial survey, DRPs that were classified as “inappropriate” were rejected and those classified as “appropriate” passed directly to the final round of discussion. DRPs classified as “uncertain” were included in a first round of panel discussion. The clinical relevance of each “uncertain” DRP was discussed and was voted on by show of hands, with at least seven votes required to pass on to the final round of discussion. All amended and new DRPs that were proposed in the survey were also discussed and rescored by each expert, using the same Likert scale. New and amended DRPs were again classified according to their median scores, and those classified as “appropriate” passed on to the final round of discussion. In the final round, all DRPs that were retained in previous steps were discussed with the perspective of selecting those that are relevant to the practice of community pharmacists. Therefore, the community pharmacists were asked to examine whether they thought the detection and management of each DRP was possible in their everyday practice. If the pharmacists answered affirmatively, a short discussion took place, followed by a vote for the inclusion of each DRP in the final list. DRPs that obtained at least seven votes were included in the final PART criteria. To determine the prevalence of DRPs in our population of KTR and test the reliability and the validity of the PART criteria, we performed a cross‐sectional study.

### 2.2 Evaluation and application of the PART criteria

#### 2.2.1 Study design, patients, and setting

In this cross‐sectional study, all KTR who had a functioning transplant for more than 1 year and were followed in a Canadian adult transplant center (CHUM) were recruited at the time of their regular follow‐up visit in the outpatient clinic between February and May 2016. Patients who were younger than 18, hospitalized, who had returned to dialysis, who had a nonrenal organ transplant, who could not provide consent or who did not speak English or French were excluded. All patients who were eligible and agreed to participate in the project were interviewed by one of the investigators (SA, QD, LER, AM) using a locally developed questionnaire. The investigator also collected data from the patient's electronic health record and obtained a detailed medication list from the patient's community pharmacy. The study was approved by the local institutional review board (CE 15.314) and written consent was obtained for each patient.

#### 2.2.2 Measurements

A complete assessment of pharmacological therapy was performed, retrieving the name and dose of each current medication used through chart review, in person patient semi‐directive interview and contact with the community pharmacy. We also collected data on age, sex, weight and height, history of previous renal transplantations, smoking and alcohol consumption, patient reported comorbidities, medication side effects, allergies, mode of medication dispensation, arterial blood pressure and capillary blood glucose values. Other laboratory results were obtained from the last available values in the year prior to the study visit in the patient's electronic health record, including serum creatinine, low‐density lipoprotein cholesterol (LDL‐C), total cholesterol, glycated hemoglobin, uric acid, total corrected calcium, serum phosphorus, and hemoglobin. Creatinine clearance was calculated using the Cockroft‐Gault formula.^13^ For each patient, all the collected information was gathered in a research file.

##### Prevalence of DRPs using the PART criteria

Once interviews were completed, the patient research files were denominalized and each file was evaluated by two independent pharmacy residents (SA, LER) who had not interviewed the patients and who had minimal experience in the field of renal transplantation. Each patient's research file was reviewed using the PART criteria to assess the number of DRPs. After they had independently coded for the presence of each DRP, the two investigators met to reach a consensus if there were discrepancies regarding their evaluation. DRPs were deemed “applicable” if they were relevant patient's clinical situations and pharmacotherapies. If a DRP was not pertinent for a given patient, it was labeled as “not applicable”. For instance, a DRP pertaining to a hypoglycemic agent in a nondiabetic patient or in a diabetic who is not using hypoglycemic agents would be labeled as “not applicable” for this patient and not be scored.

##### Prevalence of DRPs using the transplant pharmacists’ expertise

All patient research files were also independently reviewed by three expert transplant pharmacists to assess the number of DRPs using their implicit judgment and based on their clinical experience. The expert pharmacists had a baccalaureate in pharmacy, a MSc degree in advanced pharmacotherapy and a mean of 5 years of clinical experience in the field of renal transplantation. The transplant pharmacists who reviewed the patients’ research files were not involved in the development of the PART criteria and had no access to them. Conceptual validity was assessed by comparing DRPs identified by the PART criteria and those identified by the implicit judgment of the transplant pharmacists compared to all potential DRPs identified by both approaches.

#### 2.2.3 Study size and statistical analyses

We wished to build a reliable tool with substantial to almost perfect agreement. Based on our previous work,^11^ we expected a global kappa statistic of 85%. To obtain a 95% confidence interval whose full range remained in the substantial to almost perfect category (0.78‐0.90), 90 patients needed to be recruited.

Continuous variables are reported as means and standard deviations (or median and interquartile ranges where appropriate) and categorical variables are summarized as proportions. The number of patients *with at least one DRP* in each category of DRPs is reported as a proportion and 95% confidence interval. We provide both data originating from the consensus of the two evaluators (using the PART criteria) and from the transplant pharmacist's implicit judgment (not using the PART criteria).

We assessed the inter‐rater reliability of the PART criteria (whole list and each category separately) through kappa statistics, with their 95% confidence intervals. To identify patient or therapeutic characteristics associated with the number of DRPs per patient, we performed univariate and multivariate linear regression. Statistical analyses were performed using SAS version 9.3, Cary, NC, USA).

## 3 RESULTS

### 3.1 Development of the PART criteria

**Figure 1:**
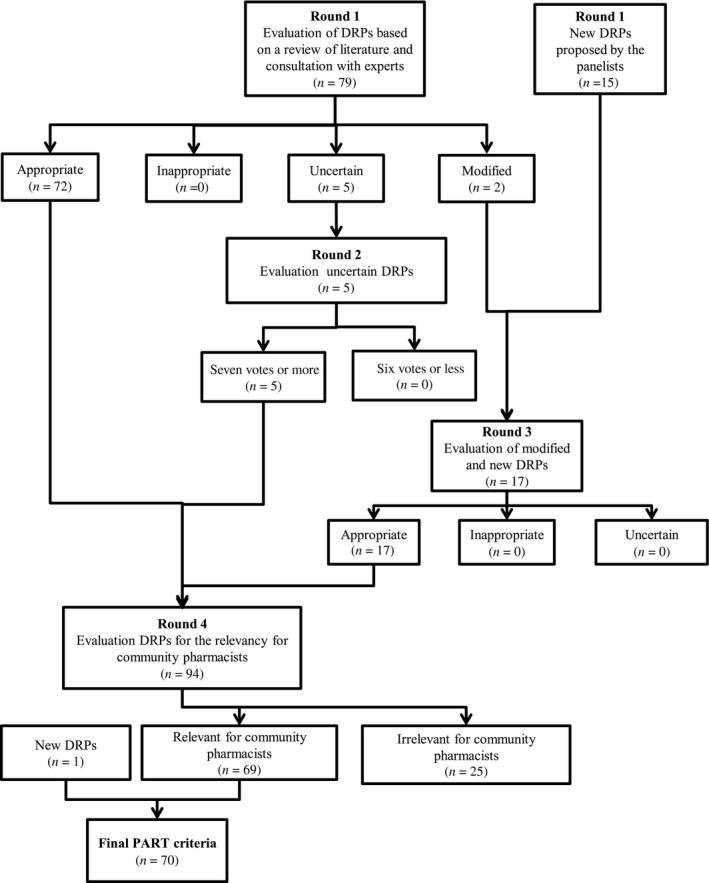
Process for defining PART (Pharmacotherapy Assessment In Renal Transplant Patients) criteria drug‐related problems using the modified RAND method

A list of 79 potential DRPs were initially sent electronically to the panel members. Figure 1 provides complete details on the steps followed in the RAND process and DRP selection. The final PART criteria included 70 DRPs (Table S1). The DRPs were classified into 10 categories: adverse events of immunosuppressant treatment, drug interactions (immunosuppressant treatment and other), nonadherence to therapy, nonoptimal blood pressure, diabetes, smoking status, phosphocalcic disorders, over the counter medications/natural products, adjustment for renal function, and other DRPs.

### 3.2 Evaluation and application of the PART criteria

We approached 99 participants between February and May 19th 2016. Two patients were excluded because they were transplanted for less than a year. The 97 other patients were recruited and included in the analysis. Patient characteristics are presented in Table 1. On average, patients were taking 11 regular medications, divided in 4 takes per day. Approximately, 58% of patients were receiving their medication in a pill box prepared by their community pharmacy. The characteristics pertaining to patient medications are presented in Table 2.

**Table 1:** Patient characteristics

Patient characteristics	n = 97
Mean age in years (standard deviation (SD))	56.7 (11.5)
Male gender, n (%)	71 (73.2)
History of previous renal transplantation, n (%)	24 (24.7)
Median time since transplantation in years (interquartile range (IQR))	9.84 (8.05)
Comorbidities	
Hypertension, n (%)	72 (74.2)
Diabetes, n (%)	24 (24.7)
Dyslipidemia, n (%)	63 (65.0)
Osteoporosis, n (%)	74 (76.3)
Anemia, n (%)	15 (15.5)
Coronary artery disease, n (%)	13 (13.4)
Gout, n (%)	17 (17.5)
Mean body mass index in kg/m^2^ (SD)	27.6 (5.1)
Mean blood pressure in mmHg	
Systolic (SD)	128 (11)
Diastolic (SD)	77.9 (7)
Laboratory values	
Mean serum creatinine in μmol/L (SD)	123 (44)
Mean estimated creatinine clearance in mL/min (SD)	62.1 (19.0)
Mean total cholesterol in mmol/L (SD)	3.60 (1.29)
Mean low‐density lipoprotein cholesterol in mmol/L (SD)	2.23 (0.80)
Mean percent glycated hemoglobin (SD)	6.1 (1.0)
Mean uric acid in μmol/L (SD)	381 (89)
Mean corrected serum calcium in mmol/L (SD)	2.40 (0.25)
Mean serum phosphorus in mmol/L (SD)	1.00 (0.19)
Mean hemoglobin in g/dL (SD)	13.4 (1.5)

**Table 2:** Characteristics of the pharmacotherapy administered (n = 97)

Mean number of total medication (standard deviation (SD))	11.8 (4.6)
Mean number of regular medications (SD)	10.8 (4.3)
Mean number of as needed medication (SD)	1.0 (1.3)
Mean number of tablets/capsules per day (SD)	15.7 (6.5)
Mean number of takes of oral medications per day (SD)	3.7 (1.3)
Mean number of takes of medication per day (including parenteral agents) (SD)	3.8 (1.4)
Medication management	
Vial, n (%)	23 (23.7)
Pill box prepared by the pharmacy, n (%)	56 (57.7)
Pill box prepared by the patient, n (%)	18 (18.6)
**Medication class**	
*Immunosuppressants*	
Calcineurin inhibitors	
Tacrolimus, n (%)	81 (83.5)
Cyclosporine, n (%)	8 (8.3)
mTOR inhibitors, n (%)	4 (4.1)
Antimetabolites	
MMF, n (%)	78 (80.4)
Azathioprine, n (%)	4 (4.1)
Leflunomide, n (%)	6 (6.2)
Oral corticosteroids, n (%)	75 (77.3)
*Antiplatelets*	
Acetylsalicylic acid, n (%)	29 (29.9)
Clopidogrel, n (%)	2 (2.1)
*Antihypertensives*	
Thiazide diuretics, n (%)	9 (9.3)
ACEi, n (%)	18 (18.6)
ARB, n (%)	24 (24.7)
DHP CCB, n (%)	47 (48.5)
Alpha blockers, n (%)	12 (12.4)
Beta blockers, n (%)	54 (55.7)
*Antihypertensives (continued)*	
Direct vasodilator, n (%)	5 (5.2)
Central agents, n (%)	5 (5.2)
*Hypoglycemic agents*	
Oral	
Metformin, n (%)	15 (15.5)
Sulfonylurea, n (%)	9 (9.3)
DPP4 inhibitors, n (%)	10 (10.3)
Parenteral	
GLP‐1 agonists, n (%)	3 (3.1)
Basal insulin, n (%)	13 (13.4)
Prandial insulin, n (%)	12 (12.4)
*Hypolipidemic agents*	
Statin, n (%)	60 (61.9)
Ezetimibe, n (%)	7 (7.2)
Fibrates, n (%)	4 (4.1)
*Phosphocalcic balance*	
Calcium supplements, n (%)	45 (46.4)
Vitamin D supplements, n (%)	71 (73.2)
Bisphosphonate, n (%)	24 (24.7)
*Management of anemia*	
Iron supplements, n (%)	11 (11.3)
EPO, n (%)	4 (4.1)
*Hypouricemic agents,* n *(%)*	18 (18.6)

ASA, acetylsalicylic acid; ACEi, angiotensin‐converting‐enzyme inhibitor; ARB, angiotensin II receptor blockers; CCB, calcium channel blocker; DHP, dihydropyridine; DPP4, dipeptidyl peptidase 4; EPO, erythropoietin; GLP‐1, glucagon‐like peptide‐1; MMF, mycophenolate mofetil; mTOR, mammalian target of rapamycin.

#### 3.2.1 Reliability of the PART criteria

A total of 115 potential DRPs were found by applying the PART criteria to all 97 patients. The average evaluation time for a single patient using the PART criteria was 6 minutes. The 97 patients included in our study were using a total of 1148 medications. Out of the latter, 873 (76%) of medications taken were targeted by the PART criteria. In 2775 occurrences, the PART criteria were deemed “applicable” and were scored as they were relevant to the patient's clinical situation and pharmacotherapy.

Two evaluators individually reviewed the patient files using the PART criteria. Each evaluator had the same number of observations (n = 2775). The global kappa statistic for the complete list of PART criteria was 0.88 (95% CI: 0.84‐0.93). The kappa statistics varied by DRP categories, ranging from 0.66 to 1.00 (Table 3). The only two categories that did not obtain a kappa above 0.8 were in the “drug interactions‐ immunosuppressant treatment and other” and “adjustment for renal function” categories (kappa statistics: 0.66 in both instances).

**Table 3:** Inter‐rater reliability of the PART criteria

Category of DRP	Inter‐rater reliability (n = 97)	
	Concordance of observations (%)	Kappa statistic (95% CI)
Adverse events of immunosuppressant treatment	278/279 (99.6)	0.95 (0.87‐1.00)
Interaction with immunosuppressant treatment	38/43 (88.4)	0.66 (0.40‐0.93)
Nonadherence to the medication therapy	331/336 (98.5)	0.90 (0.81‐0.99)
Nonoptimal arterial pressure	240/246 (97.6)	0.91 (0.84‐0.98)
Diabetes	39/40 (97.5)	0.89 (0.69‐1.00)
Smoking status	97/97 (100.0)	1.00 (NA)
Phosphocalcic disorders	2/2 (100.0)	1.00 (NA)
Over the counter medications/natural products	1355/1358 (99.8)	0.82 (0.63‐1.00)
Renal failure adjustment	184/188 (97.9)	0.66 (0.34‐0.97)
Other DRPs	186/186 (100.0)	1.00 (NA)
Total	2750/2775	0.88 (0.84‐0.93)

DRP, drug‐related problem; NA, not applicable; CI, confidence intervals.

#### 3.2.2 Prevalence of DRPs using the PART criteria

We found that 68% (95% CI: 58.8‐77.3) of patients had at least 1 DRP using the PART criteria consensus evaluation. The three most prevalent categories of DRPs were “nonoptimal blood pressure” with 42.2% (95% CI: 33.6‐53.6) of patients having at least 1 DRP in that category, while 19.6% (95% CI: 11.5‐27.5) had at least 1 DRP in the “nonadherence to therapy” category and 12.4% (95% CI: 5.8‐18.9) in the “adverse events of immunosuppressant treatment” category. The occurrence of DRPs in other categories can be found in Table 4. The average number of DRPs per patient was 1.19 (95% CI 0.97, 1.40). The maximum number of DRPs for a single patient was 4 and the minimum was 0.

**Table 4:** Prevalence of DRPs by categories using the PART consensus evaluation in 97 kidney transplant recipients

Category of DRP	Evaluations, n	DRPs detected, n	Patients with ≥1 DRP per category, n	Patients with ≥1 DRP per category (% and 95% CI)
Adverse events of immunosuppressant treatment	279	12	12	12.4 (5.8‐18.9)
Interaction with immunosuppressant treatment	43	11	8	8.2 (3.3‐13.2)
Nonadherence to therapy	336	26	19	19.6 (11.5‐27.5)
Nonoptimal arterial pressure	246	41	41	42.3 (33.6‐53.6)
Diabetes	40	6	5	5.15 (1.1‐9.2)
Smoking status	97	4	4	4.12 (0.2‐8.1)
Phosphocalcic disorders	2	0	0	—
Over the counter medications/natural products	1358	8	8	8.25 (2.8‐13.7)
Renal failure adjustment	188	7	6	6.2 (1.4‐11.0)
Other DRPs	186	0	0	—
Total (n)	2775	115	66	68.0 (58.8‐77.3)

CI, confidence interval; DRP, drug‐related problem.

#### 3.2.3 Determinants of DRPs using the PART criteria

We sought to determine whether patient or therapy‐related characteristics were associated with the number of DRPs. In multivariate analysis, the total number of medications taken was associated with the number of DRPs (β: 0.27 for an increase of five medications, 95% CI 0.005, 0.547). There were no statistically significant association between the other patient/medication characteristics and the number of DRPs (Table 5). We found no significant predictor of DRPs as identified by transplant pharmacists in regression analyses.

**Table 5:** Patient and medication characteristics associated with the number of drug‐related problems

Characteristics	β coefficient	95% confidence interval
Age (per 10‐year increase)	0.02	−0.194, 0.234
Number of medications (per 5‐medication increase)	0.27	0.005, 0.547
Number of intake per day (per 1 intake increase)	0.043	−0.144, 0.230
Time elapsed since transplantation (per 10‐year increase)	0.034	−0.347, 0.280
Male gender	−0.256	−0.754, 0.242
Dispensation mode		
Dispill	—	—
Vial	0.090	−0.617, 0.797
Dosette	−0.202	−0.789, 0.384
First transplantation	0.170	−0.354, 0.694

#### 3.2.4 Prevalence of DRPs determined by expert transplant pharmacists

To evaluate the conceptual validity of the PART criteria, transplant pharmacists who had no knowledge of or access to the PART criteria reviewed all the anonymized research files. They were asked to evaluate the presence of all clinically significant DRPs based on their best judgment. DRPs detected by transplant pharmacists and using the PART criteria consensus are presented in Table 6. Transplant pharmacists detected 52 additional DRPs that were not comprised in the PART criteria. On average, transplant pharmacists discovered 1.39 (95% CI 1.14, 1.65) DRPs per patient, with 58.8% (95% CI: 0.47‐0.68) of patients having at least 1 DRP. A total of 170 DRPs were identified by transplant pharmacists *and/or* the PART consensus evaluation. Of the latter, 138 (81%) were recognized by transplant pharmacists and 115 (67%) by the PART criteria consensus evaluation, with an overlap of 83 DRPs (49%) detected by both methods. The most frequent DRPs detected only by transplant pharmacists (n = 52) concerned simplification of the medication schedule (n = 15), nonadherence to osteoporosis prophylaxis (n = 10) and adverse effects of immunosuppressant treatment (n = 15), mostly neurotoxicity (n = 10) and abdominal pain (n = 4). Using the PART list, the unexperienced evaluators identified more PART‐DRPS in each category, leading to the identification of 29 PART‐DRPs that had not been uncovered by transplant pharmacists who had no access to the PART checklist.

**Table 6:** Number of drug‐related problems (DRPs) identified using the PART criteria and clinical judgment of renal transplant pharmacists

DRPs	PART Criteria	Transplant Pharmacists
PART‐DRPs	115	86
Adverse events of immunosuppressant treatment	12	9
Need for osteoporosis prophylaxis	4	3
Diarrhea	5	3
Hirsutism	1	1
Acne	2	2
Drug interactions (immunosuppressant treatment and other)	11	3
CNI/antifungals	2	1
CNI/antiretrovirals	1	1
Calcium/antibiotic	2	0
Calcium/iron	2	0
Phosphate chelator/levothyroxin	2	1
CNI/sevelamer	1	0
MMF/sevelamer	1	0
Nonadherence to therapy	26	22
Immunosuppressant treatment	9	8
Antihypertensive agents	6	6
Hypoglycemic agents/insulin	3	2
Lipid‐lowering drugs	4	2
Anemia management	0	1
Phosphocalcic regulators	4	3
Nonoptimal blood pressure	41	38
Patient needs antihypertensive medication	2	4
Nonoptimal antihypertensive therapy	38	34
Hypotension	1	0
Diabetes	6	1
Hypoglycemia 2nd to oral hypoglycemic agents	2	0
Hypoglycemia caused by insulin	4	1
Tobacco use	4	4
Phosphocalcic disorders	0	0
Over the counter medications/natural products	8	3
Grapefruit or grapefruit juice	1	1
Pomegranate or pomegranate juice	7	2
Adjustment for renal function	7	6
NSAID (contraindicated medication)	2	2
Elevated dose of antiviral	1	1
Nitrofurantoin (contraindicated if ClCr <60 mL/min)	2	2
Elevated dose of TMP/SMX	1	1
Bisphosphonates (contraindicated if ClCr <30‐35 ml/min)	1	0
Others	0	0
Non‐PART DRPs	0	52
Adverse events of immunosuppressive regimen		15
Abdominal pain		4
Flatulence		1
Neurotoxicity		10
Drug interactions		3^a^
Requirement for simplification of therapeutic regimen		15
Nonoptimal treatment adherence to osteoporosis prophylaxis		10
Over the counter drug use		3
Adjustment for renal function		2
Others		4
Number of DRPs	115	138

The total number of DRPs recognized by the transplant pharmacist AND/OR the PART consensus evaluation is 170 (115 PART‐DRPs identified by PART consensus, 52 non‐PART DRPs identified by transplant pharmacists, and 3 PART‐DRPs identified by transplant pharmacists and not by the PART consensus evaluation).

The 3 non‐PART drug interactions DRPs identified by transplant pharmacists were as follows: iron/levothyroxine, azathioprine/mesalamine and itraconazole/prednisone.

## 4 DISCUSSION

We developed and validated a set of criteria that can be used by the community pharmacists to assess clinically relevant DRPs in KTR. The PART criteria comprise 70 items that are highly reliable amongst observers and show good conceptual validity when compared to the implicit judgment of expert transplant pharmacists.

To develop the PART criteria, we used a modified RAND method. With this approach, it was possible to create a list of DRPs based on available evidence and clinical experience of several experts in renal transplant. A total of 70 different DRPs were included in our tool and an acceptable mean time of 6 minutes was required to evaluate the pharmacotherapy of a patient using the PART criteria. The reliability of the whole PART list was high at 0.88, with kappa statistics exceeding 0.6 for all DRP categories.^14^

The lowest kappa value (0.66) was observed for the “medication interaction” DRP category. Discordance was observed when interactions between phosphate binders/calcium and other medications (including immunosuppressants) were evaluated. The appropriate length of time required between phosphate binders/calcium and other drugs was not specified in the PART criteria. We will correct the list by specifying that a gap of less than 2 hours between phosphate binders/calcium and other medications which absorption could be decreased is considered a DRP. The low kappa value for “renal failure adjustment” DRPs was principally explained by discordance between raters relating to whether use of topical NSAIDs should be considered a DRP in this patient population. The PART list did not contain specific information on the latter. In the absence of solid data on innocuity, we will update the list and specify that NSAID use, even topical, should be considered a DRP and reported to the transplant center.

Compared to the implicit judgment of a clinical pharmacist specialized in renal transplantation, the PART list detected less DRPs on average (1.2 DRPs/patient with PART criteria vs 1.4 DRPs/patient with transplant pharmacists). However, the PART criteria detected more patients with at least 1 DRP (66 patients) than the implicit judgment of transplant pharmacists (57 patients). Most DRPs detected both using the PART criteria and by transplant pharmacists were in the suboptimal arterial blood pressure category and in the nonadherence category.

The difference in number of DRPs and the number of patients with at least 1 DRP may be explained by the fact that the pharmacists were asked to evaluate all DRPs whereas the PART criteria were limited to a set of possible DRPs, which excluded DRPs that are not relevant to community pharmacists in the development process. The PART criteria also focused on DRPs related to the kidney transplantation, and excluded general DRPs that are relevant to any other comorbidity the patient could suffer from. The DRPs detected by the renal transplant pharmacists were also related to their clinical judgment and experience which can be more subjective.

Among the DRPs detected by the transplant pharmacists, 52 were not included in the PART criteria. The most frequent DRPs detected only by transplant pharmacists concerned simplification of the medication schedule, nonadherence to osteoporosis prophylaxis, and adverse effects of immunosuppressant treatment. We regarded the decision to simplify the medication schedule for KTR as one that should be made by the transplant team, not the community pharmacist. Hence, we did not include such DRPs in the PART criteria retrospectively. As the total number of medications was associated with a greater number of DRPs, the implicit judgment of an experienced transplant pharmacist remains key to simplify the patient's regimen. This is particularly important given the high prevalence of nonadherence DRPs that we identified in our population. We will upgrade the PART criteria with DRPs for nonadherence to osteoporosis prophylaxis, abdominal and neurological side effects, as these DRPs were identified by the experienced transplant pharmacist only and could easily be evaluated and relevant for community pharmacists.

The unexperienced PART evaluators identified 29 PART‐DRPs that had not been uncovered by the explicit judgment of experienced transplant pharmacists who used no checklist to perform their evaluation. These DRPs were found in all categories. This is consistent with the improved performance associated with using checklists, an observation that has been reported in the evaluation of pharmacotherapy 15 but also in other clinical contexts such as surgery 16 and the management of acute kidney injury.^17^

The mean number of DRPs we identified in our cohort of prevalent kidney transplant recipients was less than half of the figures that our group previously reported in patients with chronic kidney disease (eGFR≤60 mL/min/1.73 m^2^) who were followed in renal protection clinics in the same region, but not the same center. Although the PART criteria were different than the ones that were elaborated for patients with CKD,^11^ we used a similar methodology to develop the transplant‐specific tool. We hypothesize that these differences are due to closer follow‐up and contact between kidney transplant recipients and their health providers when compared to patients with stage 3‐4 CKD in native kidneys.

Our study has limitations. First, the prevalence of DRPs detected in our cohort can be underestimated because we recruited patients who are regularly seen at the transplant center outpatient clinic. In the province of Quebec, this is the standard of care as most kidney transplant recipients are followed by their transplant centers and are not returned to general nephrology units while they have a functioning graft. Second, in the context of the study, the PART criteria only took 6 minutes to fill. However, because all the data necessary to apply the criteria had already been collected, the process of evaluating a patient may take a longer time in community pharmacies. This may limit the clinical usefulness of a “paper‐based” tool in the real‐life setting. As recent changes in the patients’ pharmacologic profiles were not recorded at the time of data collection, it is possible that we overestimated the prevalence of DRPs associated with hypertension control—a patient deemed not controlled could become adequately controlled within a short subsequent period if recent modifications to the antihypertensive regimen were made. However, as neither the expert transplant pharmacists nor the PART evaluators had access to this information, we do not believe that this had an impact on our evaluation of the validity or the reliability of the PART criteria.

In conclusion, the list of PART criteria represents a tool that enables detection of clinically significant DRPs by community pharmacists in KTR. Although this tool cannot replace the implicit judgment of experienced transplant pharmacists, accessibility to the latter may be limited. Community pharmacists offer proximity services to patients and are uniquely poised to detect DRPs. The PART criteria can reliably aid community pharmacists to better evaluate the pharmacotherapy of renal transplant patients and intervene in order to ensure that they have proper follow up. We are now planning on developing and validating a clinical pharmacy software integrating PART criteria‐based alarms at the level of community pharmacies to assess the acceptability and usefulness of this tool in the real‐life setting.

## AUTHOR CONTRIBUTIONS

NB, EGL, HC, and LL contributed to research idea and study design. SA, QD, AM, and LER involved in data acquisition. SA and LER involved in detection of DRPs based on PART criteria. SA, QD, AM, LER, NB, HC, and LL involved in data analysis/interpretation. DB, QD, AM, and HC involved in statistical analysis. SA, QD, LER, and AM drafted initial manuscript. DB, NB, HC, and LL reviewed and revised manuscript. NB, and HC involved in supervision or mentorship. Each author contributed important intellectual content during manuscript drafting or revision and accepts accountability for the overall work by ensuring that questions pertaining to the accuracy or integrity of any portion of the work are appropriately investigated and resolved.

## DISCLOSURE

El Raichani, Layal : none. Du, Qian: none. Mathieu, Alexandre : none. Almassy, Sabrina: none. Lyne Lalonde : none. Gélinas‐Lemay, Elisabeth: none. Boudreau, Nathalie: none. Cardinal, Héloïse: Innovation Grants from Astellas‐CNTRP 2016, 2017 (unrelated to the current study, total of 50, 000.00$).
