# Supplementary material for: Development and validation of PART (Pharmacotherapy Assessment in Renal Transplant Patients) criteria to assess drug‐related problems in an outpatient renal transplant population: A cross‐sectional study
Source: Pharmacol Res Perspect. 2019 Jan 15;7(1):e00453. doi: 10.1002/prp2.453 (PMC6333916; doi:10.1002/prp2.453)
Supplement: Supplementary file 1 [file PRP2-7-e00453-s001.docx]

**Supplementary Table 1. PART criteria**

| # | DRP | Please write YES/NO or non applicable (N/A) |
| --- | --- | --- |
| **Adverse events of immunosuppressant treatment** | |  |
| 1 | The patient is receiving high dose corticosteroids and is not receiving a prophylaxis for osteoporosis. |  |
| 2 | The patient is suffering from diarrhea secondary to renal transplant. |  |
| 3 | The patient is suffering from hirsutism secondary to cyclosporine use. |  |
| 4 | The patient is suffering from acne secondary to anti-rejection medication use. |  |
| **Drug interactions (immunosuppressant treatment and other)** | |  |
| 5 | Presence of an interaction between calcineurin inhibitors and antifungals. |  |
| 6 | Presence of an interaction between mTOR inhibitors and antifungals. |  |
| 7 | Presence of an interaction between calcineurin inhibitors and macrolides. |  |
| 8 | Presence of an interaction between mTOR inhibitors and macrolides. |  |
| 9 | Presence of an interaction between calcineurin inhibitors and antiretrovirals. |  |
| 10 | Presence of an interaction between mTOR inhibitors and antiretrovirals. |  |
| 11 | Presence of an interaction between calcineurin inhibitors and non-dihydropyridine calcium channel blockers. |  |
| 12 | Presence of an interaction between mTOR inhibitors and non-dihydropyridine calcium channel blockers. |  |
| 13 | Presence of an interaction between calcineurin inhibitors and certain anticonvulsants (carbamazepine, phenytoin, phenobarbital). |  |
| 14 | Presence of an interaction between mTOR inhibitors and certain anticonvulsants (carbamazepine, phenytoin, phenobarbital). |  |
| 15 | Presence of an interaction between calcineurin inhibitors and rifampin. |  |
| 16 | Presence of an interaction between mTOR inhibitors and rifampin. |  |
| 17 | There is presence of a medication interaction between sevelamer and calcineurin inhibitor. |  |
| 17 | Presence of an interaction between calcium carbonate and an antibiotic (tetracycline or quinolone). |  |
| 18 | Presence of an interaction between calcium carbonate and iron supplement. |  |
| 19 | The patient is not taking phosphate binders (calcium carbonate, sevelamer or lanthanum) appropriately. |  |
| 20 | There is presence of a medication interaction between phosphate binders (calcium carbonate, sevelamer or lanthanum) and levothyroxine. |  |
| 21 | Presence of an interaction between sevelamer and calcineurin inhibitor. |  |
| 22 | Presence of an interaction between sevelamer and mycophenolate mofetil. |  |
| 23 | Presence interaction between sevelamer or lanthanum and ciprofloxacine |  |
| 24 | Presence of an interaction between azathioprine and xanthine oxidase inhibitor. |  |
| 25 | The dosage of colchicine used for the treatment or prophylaxis of gout flare is too elevated because of an interaction with cyclosporine. |  |
| **Non-adherence to therapy** | |  |
| 26 | The patient is not adherent to immunosuppressant treatment (patient is using less than 90% the usual amount of medication during a period of 90 days). |  |
| 27 | The patient is not adherent to antihypertensive treatment (patient is using less than 80% the usual amount of medication during a period of 90 days). |  |
| 28 | The patient is not adherent to diabetic treatment (patient is using less than 80% the usual amount of medication during a period of 90 days). |  |
| 29 | The patient is not adherent to dyslipidemia treatment (patient is using less than 80% the usual amount of medication during a period of 90 days). |  |
| 30 | The patient is not adherent to anemia treatment (patient is using less than 80% the usual amount of medication during a period of 90 days). |  |
| 31 | The patient is not adherent to phosphocalcic metabolism regulating treatment (patient is using less than 80% the usual amount of medication during a period of 90 days) |  |

| **Non optimal blood pressure** |  |
| --- | --- |

| 32 | The patient needs antihypertensive medication because his arterial pressure is superior to 130/80 mmHg but is not receiving any such therapy. |  |
| --- | --- | --- |
| 33 | The patient receives a sub-optimal antihypertensive treatment because his arterial pressure is superior to 130/80 mmHg despite current therapy. |  |
| 34 | The patient developed hypotension, symptoms of hypotension or orthostatic hypotension secondary to the antihypertensive agents he/she is receiving. |  |
| **Diabetes** | |  |
| 35 | The patient experiences adverse effects (hypoglycemia) after taking hypoglycemic agents. |  |
| 36 | The patient experiences adverse effects (hypoglycemia) after taking insulin. |  |
| **Smoking status** | |  |
| 37 | The patient is a smoker, but no intervention was undertaken to favor a reflection on his smoking status, he/she is not receiving a nicotine replacement therapy or another smoking cessation treatment. |  |
| **Phosphocalcic disorders** | |  |
| 38 | The patient is receiving an aluminum based phosphate binder for more than 3 months consecutively. |  |
| **Over the counter medications/natural products** | |  |
| 39 | The patient is consuming grapefruit or grapefruit juice while taking immunosuppressants metabolized by cytochrome 3A4. |  |
| 40 | The patient is taking echinacea while taking an immunosuppressant treatment. |  |
| 41 | The patient is taking Saint John’s wort while taking an immunosuppressant treatment. |  |
| 42 | The patient is taking ginseng while taking an immunosuppressant treatment. |  |
| 43 | The patient is taking uncaria tomentosa (cat’s claw) while taking an immunosuppressant treatment. |  |
| 44 | The patient is taking melatonin while taking an immunosuppressant treatment. |  |
| 45 | The patient is taking antacids containing magnesium or aluminum while taking mycophenolate mofetil. |  |
| 46 | The patient is taking garlic supplements while taking an immunosuppressant metabolized by cytochrome 3A4. |  |
| 47 | The patient is taking peppermint oil while taking an immunosuppressant metabolized by cytochrome 3A4. |  |
| 48 | The patient is taking ginkgo biloba while taking an immunosuppressant metabolized by cytochrome 3A4. |  |
| 49 | The patient is taking arabinogalactan while taking an immunosuppressant treatment. |  |
| 50 | The patient is taking pomegranate or pomegranate juice while taking an immunosuppressant metabolized by cytochrome 3A4. |  |
| 51 | The patient is taking kava while taking an immunosuppressant metabolized by cytochrome 3A4. |  |
| 52 | The patient is taking liquorice while taking an immunosuppressant metabolized by cytochrome 3A4. |  |
| **Adjustment for renal function** | |  |
| 53 | The patient is receiving a NSAID, which is contraindicated after renal transplantation |  |
| 54 | The patient is receiving a dose of gabapentin that is too high for his renal function. |  |
| 55 | The patient is receiving a dose of pregabalin that is too high for his renal function. |  |
| 56 | The patient is receiving a dose of antiviral (acyclovir, valacyclovir, famciclovir) that is too high for his renal function. |  |
| 57 | The patient is receiving a dose of cephalosporin that is too high for his renal function. |  |
| 58 | The patient is receiving a dose of neuraminidase inhibitor (eg. oseltamivir) that is too high for his renal function. |  |
| 59 | The patient is receiving nitrofurantoin, which is contraindicated in chronic kidney diseases (CrCl < 60 mL/min). |  |
| 60 | The patient is receiving a dose of quinolone (ciprofloxacin or levofloxacin) that is too high for his renal function. |  |
| 61 | The patient is receiving a dose of TMP-SMX that is too high for his renal function. |  |
| 62 | The patient is receiving a dose of antifungal (eg. fluconazole) that is too high for his renal function. |  |
| 63 | The patient has a creatinine clearance inferior to 25 mL/min and is receiving a contraindicated medication, acarbose. |  |
| 64 | The patient has a creatinine clearance inferior to 30 mL/min and is receiving a contraindicated medication, metformin. |  |
| 65 | The patient is receiving a dose of ranitidine that is too high for his renal function. |  |
| 66 | The patient has a creatinine clearance inferior to 30-35 mL/min and is receiving a contraindicated medication, an oral bisphosphonate. |  |
| 67 | The patient is receiving a dose of NOAC (dabigatran, rivaroxaban, apixaban) that is too high for his renal function. |  |
| **Other DRPs** | |  |
| 68 | The patient does not apply sun protection. |  |
| 69 | The patient is taking Advagraf BID while Prograf BID was prescribed or the patient is taking Prograf die while Advagraf die was prescribed. |  |
| 70 | A change of generic was necessary for a medication with a narrow therapeutic index (notably tacrolimus). |  |
